# Enhanced Cytotoxicity and Antimelanoma Activity of Novel Semisynthetic Derivatives of Betulinic Acid with Indole Conjugation

**DOI:** 10.3390/plants13010036

**Published:** 2023-12-21

**Authors:** Adelina Lombrea, Claudia Geanina Watz, Larisa Bora, Cristina Adriana Dehelean, Zorita Diaconeasa, Stefania Dinu, Māris Turks, Jevgeņija Lugiņina, Uldis Peipiņš, Corina Danciu

**Affiliations:** 1 Department of Pharmacognosy, “Victor Babes” University of Medicine and Pharmacy Timisoara, Eftimie Murgu Square, No. 2, 300041 Timisoara, Romania; adelina.lombrea@umft.ro (A.L.); larisa.bora@umft.ro (L.B.); corina.danciu@umft.ro (C.D.); 2 Research Center for Pharmaco-Toxicological Evaluation, “Victor Babes” University of Medicine and Pharmacy Timisoara, Eftimie Murgu Square, No. 2, 300041 Timisoara, Romania; cadehelean@umft.ro; 3 Department of Pharmaceutical Physics, Faculty of Pharmacy, “Victor Babes” University of Medicine and Pharmacy of Timisoara, 300041 Timisoara, Romania; 4 Department of Toxicology and Drug Industry, Faculty of Pharmacy, “Victor Babes” University of Medicine and Pharmacy Timisoara, 300041 Timisoara, Romania; 5 Department of Biochemistry, University of Agricultural Sciences and Veterinary Medicine, 400372 Cluj-Napoca, Romania; zorita.sconta@usamvcluj.ro; 6 Department of Biotechnology, BIODIATECH—Research Centre for Applied Biotechnology in Diagnosis and Molecular Therapy, 400478 Cluj-Napoca, Romania; 7 Department of Pedodontics, Faculty of Dental Medicine, “Victor Babes” University of Medicine and Pharmacy Timisoara, 9 No., Revolutiei Bv., 300041 Timisoara, Romania; dinu.stefania@umft.ro; 8 Pediatric Dentistry Research Center, Faculty of Dental Medicine, “Victor Babes” University of Medicine and Pharmacy Timisoara, 9 No., Revolutiei Bv., 300041 Timisoara, Romania; 9 Institute of Technology of Organic Chemistry, Faculty of Materials Science and Applied Chemistry, Riga Technical University, P. Valdena Str. 3, LV-1048 Riga, Latvia; maris.turks@rtu.lv (M.T.); jevgenija.luginina@rtu.lv (J.L.); sales@nstchemicals.com (U.P.); 10 Nature Science Technologies Ltd., Rupnicu Str. 4, LV-2114 Olaine, Latvia

**Keywords:** 2,3-indolo-betulinic acid, glycine conjugates, melanoma, B164A5 murine melanoma cells

## Abstract

The prevalence and severity of skin cancer, specifically malignant melanoma, among Caucasians remains a significant concern. Natural compounds from plants have long been explored as potential anticancer agents. Betulinic acid (BI) has shown promise in its therapeutic properties, including its anticancer effects. However, its limited bioavailability has hindered its medicinal applications. To address this issue, two recently synthesized semisynthetic derivatives, *N*-(2,3-indolo-betulinoyl)diglycylglycine (BA1) and *N*-(2,3-indolo-betulinoyl)glycylglycine (BA2), were compared with previously reported compounds *N*-(2,3-indolo-betulinoyl)glycine (BA3), 2,3-indolo-betulinic acid (BA4), and BI. These compounds were evaluated for their effects on murine melanoma cells (B164A5) using various in vitro assays. The introduction of an indole framework at the C2 position of BI resulted in an increased cytotoxicity. Furthermore, the cytotoxicity of compound BA4 was enhanced by conjugating its carboxylic group with an amino acid residue. BA2 and BA3, with glycine and glycylglycine residues at C28, exhibited approximately 2.20-fold higher inhibitory activity compared to BA4. The safety assessment of the compounds on human keratinocytes (HaCaT) has revealed that concentrations up to 10 µM slightly reduced cell viability, while concentrations of 75 µM resulted in lower cell viability rates. LDH leakage assays confirmed cell membrane damage in B164A5 cells when exposed to the tested compounds. BA2 and BA3 exhibited the highest LDH release, indicating their strong cytotoxicity. The NR assay revealed dose-dependent lysosome disruption for BI and 2,3-indolo-betulinic acid derivatives, with BA1, BA2, and BA3 showing the most cytotoxic effects. Scratch assays demonstrated concentration-dependent inhibition of cell migration, with BA2 and BA3 being the most effective. Hoechst 3342 staining revealed that BA2 induced apoptosis, while BA3 induced necrosis at lower concentrations, confirming their anti-melanoma properties. In conclusion, the semisynthetic derivatives of BI, particularly BA2 and BA3, show promise as potential candidates for further research in developing effective anti-cancer therapies.

## 1. Introduction

Since ancient times, humans have recognized and utilized natural substances as the primary source of therapeutic medications [1]. The late 19th and early 20th centuries were characterized by breakthroughs in organic chemistry and chemical analysis, thus paving the way for the isolation, purification, and characterization of several bioactive substances produced by plants [2,3]. Consequently, numerous natural chemicals obtained from plants have been used as the primary raw resources for a variety of medications. Innovative medicinal properties of plant secondary metabolites have been recognized by contemporary phytotherapy. According to a large number of studies published to date, phytochemicals, including alkaloids, flavonoid terpenoids, and carotenoids, etc., exhibit antidiuretic, anti-inflammatory, anti-analgesic, anticancer, anti-viral, antimalarial, anti-bacterial, and anti-fungal effects and have a significant impact in controlling a variety of illnesses [4,5,6,7]. Currently, over 40% of the medications that have received approval from the FDA in the United States are derived from plants [8]. It has been witnessed that plant-based pharmaceutical and cosmetic products are becoming increasingly popular daily.

Terpenes are one of the most extensively researched classes of secondary metabolites. Terpenes, and triterpenes in particular, hold a massive role in this chemical realm, and their research has risen substantially within the previous decade [9]. Notably, their promise for cancer treatment has been acknowledged [10,11,12,13]. Pentacyclic triterpenoid carboxylic acids are of special relevance within the class of triterpenoids. In recent years, various research groups successfully characterized a large number of derivatives, focusing mostly on derivatives of betulinic, betulonic, ursolic, oleanolic, glycyrrhetinic, maslinic, and asiatic acids. Chemical modifications are recognized as milestones in achieving strong cytotoxic activity while maintaining high selectivity [14,15,16,17,18].

Betulin, betulinic acid, and betulonic acid are pentacyclic triterpenoids found in over 200 plant species [19,20]. Betulinic acid, betulonic acid, and their derivatives possess a wide range of pharmacological effects, including anti-HIV, anti-parasitic, anti-inflammatory, and anti-tumor effects [20,21,22,23,24,25,26]. Since betulinic acid is poorly soluble in aqueous solutions, it is frequently derivatized to promote solubility and improve the pharmacological activity [27,28,29]. Bevirimat, one of the most well-known analogs of betulinic acid, has been shown to suppress HIV via a unique method of action known as viral maturation inhibition. This anti-HIV agent completed Phase IIb clinical trials [25]. Although betulin is readily accessible and can be separated from the bark of birch trees, betulinic acid has a higher bioactivity. Betulinic acid is commonly derived from betulin by simple oxidation-reduction methods because it is acquired in small amounts from natural sources. In 1995, it was discovered that betulinic acid induces apoptosis in human melanoma cells, resulting in a significant suppression of these cells [30]. In addition to inducing apoptosis in A375 human melanoma cells, betulinic acid administration at a sub-toxic dosage (10 µM) might cause mitochondrial malfunction by reversing the loss in mitochondrial potential and altering mitochondrial shape [31]. In addition, it is capable of inhibiting the activity of the stress transcription factor NF-κB [32]. One other method of inhibiting tumor development is the total or partial suppression of angiogenesis [33]. Subsequent investigations have shown that the antiangiogenic impact is accomplished by mitochondrial regulation [34]. Moreover, the betulinic acid structure permits structural modifications to the functional groups at the C-3, C-28, C-20, C-30, and C-17 sites, leading to the generation of derivatives with enhanced antitumor and pharmacokinetic features [35,36,37,38]. For instance, considerable antitumor potency was discovered in betulinic acid derivatives with indolyl substitutions at sites C-2 and C-3. The inclusion of a chlorine molecule at the C-5 site of the indole ring was reported to improve activity against MIAPaCa, PA-1, and SW620 cells (IC_50_ = 2.44–2.50 µg/mL) [39]. A recent study has assessed the cytotoxic potential of novel betulinic acid−1,2,4−triazole derivatives against human malignant melanoma cells RPMI−7951. The results have shown increased cytotoxicity of the derivatives compared to betulinic acid (IC_50_ = 18.8 μM–20.7 μM). Moreover, betulinic acid derivatives triggered apoptosis-related nuclear alterations and mitochondrial dysfunction [36]. Yang et al. synthesized a series comprising betulinic acid ester derivatives and showed their strong antiproliferative potential with a proapoptotic effects against MGC-803 (human gastric cancer cell line), PC3 (human prostate carcinoma cell line), Bcap-37 (human breast carcinoma cell line), A375 (human malignant melanoma cell line), and MCF-7 (human breast carcinoma cell line) [40].

Being one of the top causes of mortality and a key impediment to improving life expectancy, cancer has a significant influence on populations across the globe. In 2020, a predicted 19.3 million novel diagnoses of cancer and about 10 million deaths were attributed to cancer [41]. Despite the tremendous development of new medicines, the resilience of cancer cells and the severe side effects of the medications employed continue to pose the greatest obstacles to medical progress [42]. With an annual recurrence rise of 0.6% malignant melanoma is considered to be the most severe subtype [43]. Melanoma is a malignant tumor that emerges from neuroectodermal melanocytic cells. As melanocytes are located in several locations throughout the body, melanoma may arise in numerous sites. The most frequent location is the skin, although it may also be located in the digestive system, urogenital tract, mucous membranes, meninges, and eyes. UVB light exposure is the primary factor responsible for melanoma onset [44]. Surgical removal of early-stage melanoma is very beneficial, but when the cancer has progressed to the point where distant metastases are present, the 5-year survival rate plummets to 1–2%, and the median survival period is just 6–9 months [45]. In conjunction with surgical treatments, chemotherapeutic and immunotherapeutic procedures are now used to treat patients. Although novel melanoma therapies have been developed in recent years, researchers are currently searching for other therapy approaches to enhance therapeutic results and prolong patient lives [46]. Hence, there is an ongoing requirement for the development of novel anti-cancer medications. Natural compounds originating from plants have been the principal source of oncological drugs and continue to offer novel anticancer agents [47].

In view of these encouraging findings, this study was designed in order to determine how a combination of fused heterocyclic moiety C2/3 and a peptide chain at C28 positions affects the cytotoxicity, cell viability, anti-migratory, and nuclear alterations of betulinic acid scaffold against the murine melanoma cell line B164A5.

## 2. Results

### 2.1. Compounds Used in the Study

**Figure 1:**
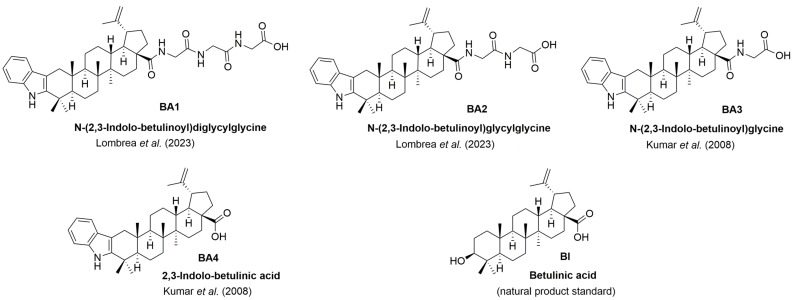
Betulinic acid derivatives used in this study; for synthesis of BA1 and BA2, see [48]; for synthesis of BA3 and BA4, see [39]. Figure adapted from [48].

Betulinic acid derivatives recently reported by us-*N*-(2,3-indolo-betulinoyl)diglycylglycine (BA1) and *N*-(2,3-indolo-betulinoyl)glycylglycine (BA2) [48]-were compared with betulinic acid (BI) as natural product standard and *N*-(2,3-indolo-betulinoyl)glycine (BA3), and 2,3-indolo-betulinic acid (BA4) that were previously reported by Kumar and Jaggi in 2008 (Figure 1) [39]. Synthesis and characterization of all compounds used in this study can be found in our earlier research paper [48].

### 2.2. Cell Viability Assay

#### 2.2.1. B164A5

**Figure 2:**
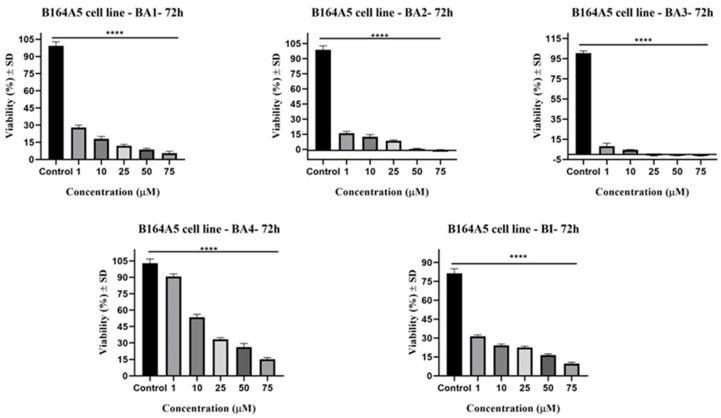
The cytotoxicity of compounds BI, BA1, BA2, BA3, and BA4 on the B164A5 cell line was assessed by conducting the MTT (3-(4,5-dimethylthiazol-2-yl)-2,5-diphenyltetrazolium bromide) assessment after a 72-h treatment period. at varied concentrations (1, 10, 25, 50, and 75 µM) of the screened compounds. The groups were subjected to a one-way analysis of variance (ANOVA) and then analyzed using Dunnett’s post-test for multiple comparisons. A *p*-value below 0.05 was deemed to be statistically significant (**** *p* ≤ 0.0001 compared to the control group).

The cytotoxicity of compounds BI, BA1, BA2, BA3, and BA4 on the B164A5 cell line was assessed by conducting the MTT (3-(4,5-dimethylthiazol-2-yl)-2,5-diphenyltetrazolium bromide) assessment after a 72-h treatment period at varied concentrations (1, 10, 25, 50, and 75 µM) of the screened compounds. The use of this approach facilitated the determination of IC_50_ values, as shown in Table 1. The observed cell viability in the examined cell line is illustrated in Figure 2. All five compounds demonstrated a significant reduction in cell viability compared to the control group, with statistical significance (*p* < 0.0001). The cellular viability was seen to be substantially diminished even at lower doses of BA1 and BA2 (1 µM), resulting in reductions of 27% and 15%, respectively, as compared to BI (32%). The derivatives of 2,3-indolo-betulinic acid exhibited reduced IC_50_ values against the B164A5 cell line in comparison to their precursor compound (Table 1). The compounds BA3 and BA2 demonstrated the highest cytotoxicity, with IC_50_ values of 8.11 μM and 9.15 μΜ, respectively.

#### 2.2.2. HaCaT

**Figure 3:**
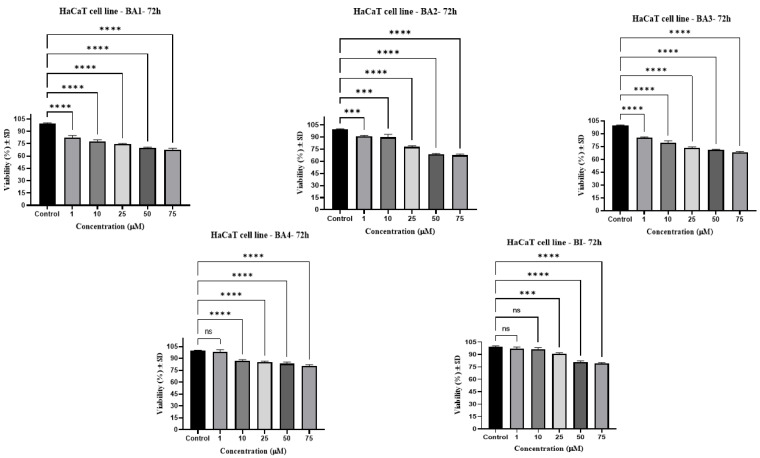
The cytotoxicity of compounds BI, BA1, BA2, BA3, and BA4 on the HaCaT cell line was evaluated using the MTT (3-(4,5-dimethylthiazol-2-yl)-2,5-diphenyltetrazolium bromide) assay after a 72 h treatment period. The compounds were tested at different concentrations (1, 10, 25, 50, and 75 µM). The groups underwent a one-way analysis of variance (ANOVA) and were then examined using Dunnett’s post-test for multiple comparisons. A *p*-value less than 0.05 was considered to have statistical significance (*** *p* ≤ 0.001; **** *p* ≤ 0.0001; ns (non-significant) compared to the control group).

The effect of testing substances (BI and BA1-BA4) on the survival of human keratinocytes cell line HaCat was also assessed. The development of melanoma is highly associated with these specific cell types [49]. As illustrated in Figure 3, the viability of normal HaCaT cell lines was reduced by nearly 22% when exposed to betulinic acid derivatives at concentrations of up to 10 µM compared to the control group. In addition, increasing dosages (25, 50, and 75 μM) caused a gradual reduction in cell viability, resulting in a decline of up to 32% for both BA1, BA2, and BA3. Significantly, BA4 and BI exhibited reduced cell toxicity at greater doses, with a decrease of around 21%.

### 2.3. Evaluation of the Cytotoxic Potential by Lactate Dehydrogenase (LDH) Assay

**Figure 4:**
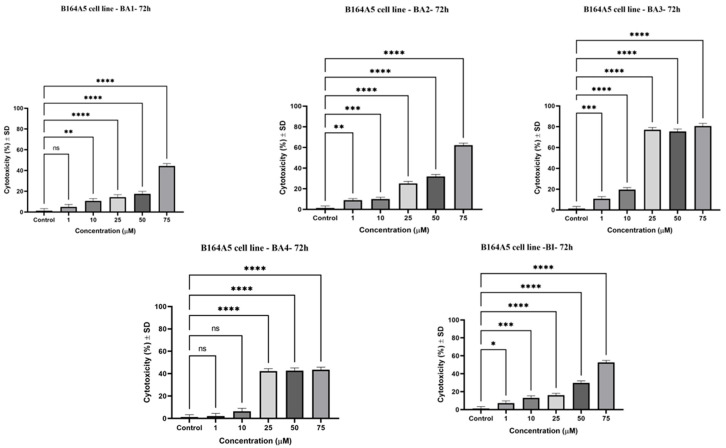
The LDH release ratio of B164A5 cells after exposure to varying doses of BA1–BA4 and BI (1, 10, 25, 50, and 75 µM) over a duration of 72 h. The statistical distinctions were assessed by the use of one-way (ANOVA) complemented by Dunnett’s multiple comparisons test (* *p* ≤ 0.05; ** *p* ≤ 0.01; *** *p* ≤ 0.001; **** *p* ≤ 0.0001; ns (non-significant) against control cells).

Figure 4 illustrates the proportion of LDH leakage, which indicates the loss of cell membrane integrity, in murine melanoma cells (B164A5) after 72 h exposure to BA1, BA2, BA3, BA4, and BI. The amount of extracellular LDH and the analyzed chemicals had a concentration-dependent interaction, as shown in Figure 4. Among the derivatives, BA2 and BA3 demonstrated the highest degree of LDH release (62.14% and 80.68%, respectively) at a concentration of 75 µM, in comparison to BI (52.7%). The findings obtained using the LDH release technique are consistent with the data obtained from the MTT assay (Figure 2 and Table 1), indicating a similar cytotoxic pattern for the tested substances.

### 2.4. Cytotoxic Effects by Means of the Neutral Red (NR) Assay

**Figure 5:**
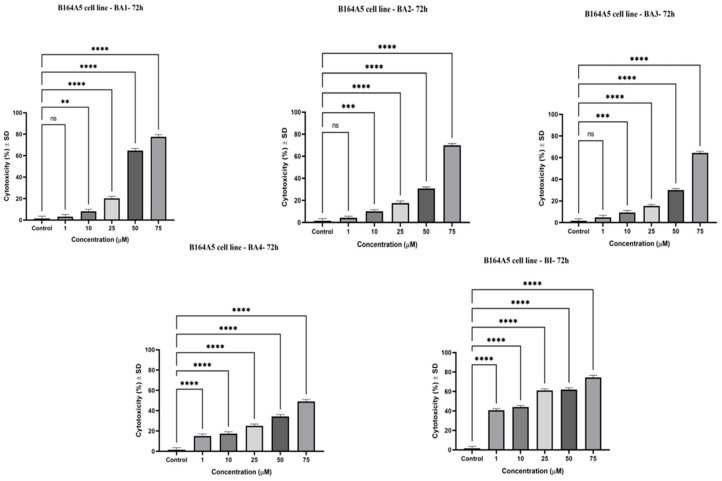
Cytotoxic extent of murine melanoma cells (B164A5) when exposed to varying doses of BA1–BA4 and BI over a period of 72 h. The data are expressed in terms of mean ± SD. The statistically significant variations were determined using one-way ANOVA analysis, subsequently followed by Dunnett’s comparisons test (** *p* ≤ 0.01; *** *p* ≤ 0.001; **** *p* ≤ 0.0001; ns (non-significant) against control cells).

The assessment of sample-induced cytotoxicity, namely the disruption of lysosomes, was conducted using the NR assay. The cells exhibited elevated cytotoxicity, which was shown to be dose-dependent on the administration of BI and 2,3-indolo-betulinic acid derivatives (Figure 5). Particularly noteworthy is the fact that BA1 exhibited a substantial cytotoxic effect at a concentration of 75 µM, resulting in a cytotoxicity level of 77.5%. When 75 µM concentrations of BA2 and BA3 samples were applied for a period of 72 h, the cytotoxic reactions exhibited comparable increases in percentages, with recorded values of 69.9% and 64.2%, respectively. Additionally, it appears that the enhanced cytotoxic effect of BA2 and BA3, as assessed using the NR and LDH assessments, had a significant correlation with the decreased cell viability rate of B164A5 at equivalent dosages.

### 2.5. Anti-Migratory Activity Evaluation Employing the Scratch Assay Method

**Figure 6:**
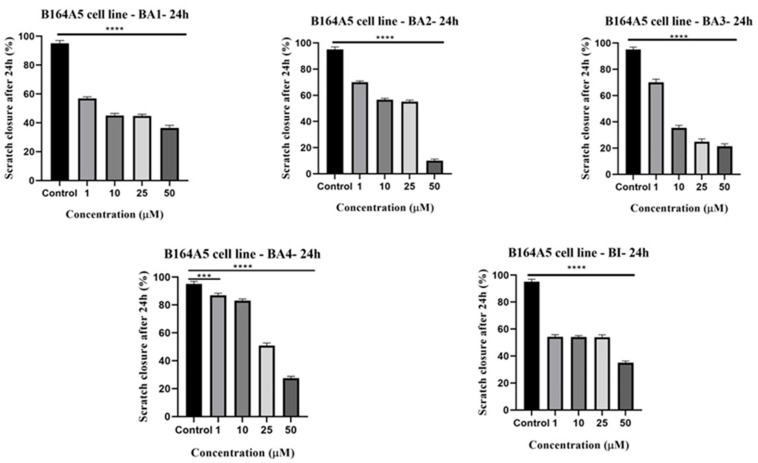
The provided images illustrate the migratory capacity of B164A5 cells after a 24 h treatment with BA1–BA4 and BI. The presented bar graphs illustrate the proportional representation of wound closure in relation to the initial surface area after a 24 h period. The findings are presented as the average values ± SD of three distinct experiments conducted in triplicate. The study used the one-way ANOVA followed by Dunnett’s multiple comparisons post-test to assess the presence of significant differences between the control group and the treatment groups. Statistical significance was denoted by *** (*p* ≤ 0.001), and **** (*p* ≤ 0.0001) when compared to the control group.

The investigation of the putative anti-migratory effect of BI and 2,3-indolo-betulinic acid derivatives has been conducted using a wound healing test, considering the significant metastatic behavior shown by malignant melanoma cells. For this evaluation, the maximum concentrations (75 µM) that caused substantial cytotoxicity in the B164A5 cell line (Figure 2) were disregarded due to the fact that it might have induced a very important cell viability decrease and no migratory activity would have been quantified. The effects exerted by the investigated compounds on B164A5 cells were highly concentration-dependent (Figure 6). The highest observed inhibition of migratory activity was seen in the group treated with BA2 at a concentration of 50 µM, resulting in a wound healing rate of 10.0%. This was closely followed by the group treated with BA3 at a concentration of 50 µM, which exhibited a wound healing rate of 21.3%. In contrast, at the same concentration, the parent compound BI inhibited the migratory capacity of melanoma cells by achieving 34.9% wound healing rates. Furthermore, the visual evidence provided in Supplementary Figures S1–S3 demonstrates that after 24 h of exposure to the tested compounds, cells exhibited apoptotic characteristics, such as changes in shape and morphology, as well as cell disintegration (refer to Supplementary Figures S1–S3). The aforementioned findings suggest that the 2,3-indolo-betulinic acid derivatives had lethal effects on B165A5 cells.

### 2.6. Nuclear Staining Using Hoechst 33342

**Figure 7:**
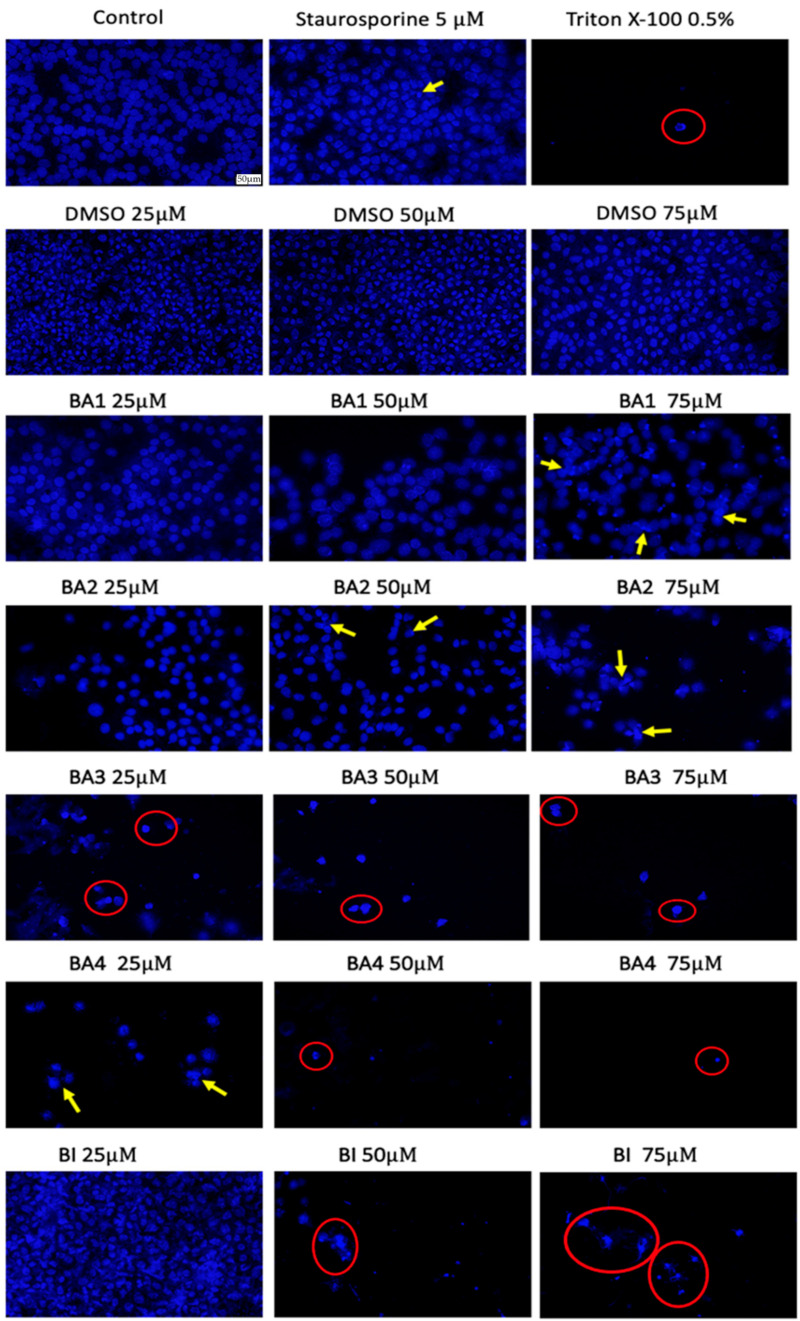
Staining of cell nuclei of murine melanoma B164A5 cells using Hoechst 33342 reagent after 72 h exposure to three different concentrations (25, 50, 75 μM) of BA1-BA4 and BI. Staurosporine at concentration of 5 μM was used as positive control for apoptosis features and Triton X 100 at concentration of 0.5% was employed to observe the necrosis process. The apoptosis-related changes are marked by yellow arrows, whereas necrosis is highlighted by a red circle. The scale bar represents 50 µm.

The evaluation of the cell nuclei morphology was realized by means of Hoechst 3342 staining in order to provide a more complex insight on the mechanism of action of the test samples (BA1, BA2, BA3, BA4 and BI). Also, the solvent-DMSO was tested in order to exclude any possible cytotoxic influence related to the solvent. Based on the results obtained for the cell viability and cytotoxicity tests, for this evaluation, only the highest concentrations were selected (25, 50, 75 μM), where an important cell functionality impairment was recorded. Thus, as depicted in Figure 7, DMSO-treated cells presented no signs of apoptosis or necrosis; the BA1 sample induced apoptosis features only at a concentration of 75 μM, while the cells treated with BA2 exhibited specific signs of apoptosis (chromatin condensation) at both concentrations of 50 and 75 μM. Nevertheless, the BA3 sample induced the most important nuclear changes, the necrosis process being observed from concentration of 25 μM, while the cells exposed to BA4 and BI manifested necrosis only from a concentration of 50 μM.

## 3. Discussion

Betulinic acid (BI) is a bioactive molecule derived from plants, with significant therapeutic promise in the treatment of several disorders [50]. Its many actions, such as antibacterial, antiviral, anti-inflammatory, and anticancer properties, demonstrate the multifunctional nature of this compound. BI has shown significant cytotoxic activity against several forms of cancer both in vitro and in vivo, indicating its potential as an effective treatment for cancer [51]. However, when considering medicinal applications, the use of BI as a possible therapeutic agent is hampered by its restricted solubility and diminished bioavailability in vivo [52]. Therefore, BI has been used as a scaffold for several semisynthetic derivatives that exhibit promise as enhanced pharmaceutical agents [36,53,54,55,56,57,58,59,60]. The indole scaffold has significant importance within the domains of medical chemistry and organic chemistry [61,62,63,64]. According to reports, indole derivatives have been shown to have allosteric characteristics in relation to a range of G protein-coupled receptors (GPCRs). This suggests that these chemicals have the ability to interact with GPCRs and elicit alterations in their structure or conformational transitions, thereby impacting their signal transduction and overall functionality [65,66]. The aforementioned findings have significant implications for the advancement of GPCR modulators and their potential therapeutic applications in the management of many ailments, including neurological diseases and cancer [61].

In the context of our prior research, we have recently reported synthesis of compounds BA1/BA2 and biological activity studies thereof [48]. The latter included the determination of antiproliferative, cytotoxic, and anti-migratory effects towards the A375 human melanoma cell line. The obtained data were evaluated in comparison with the previously known compounds BA4 and BA3 [39].

The objective of this research paper was to evaluate the antiproliferative, cytotoxic, anti-migratory, and pro-apoptotic properties of the four BI derivatives and BI on murine melanoma cells (B164A5). The intention is to subsequently investigate their potential as anti-melanoma agents in vivo using a murine melanoma animal model. The study found that the cell viability of A375 human melanoma cells decreased in a dose-dependent manner after 72 h of incubation with 2,3-indolo-betulinic acid derivatives substituted with glycine moieties. Three out of four derivatives of 2,3-indolo-betulinic acid exhibited enhanced antiproliferative activity against A375 cells, with the highest potential being BA1, which showed an IC_50_ value of 5.7 µM. The most substantial decline in the percentage of viable cells was reported at the highest doses examined (25, 50, and 75 µM). The tested derivatives exhibited an important decline in the percentage of viable cancer cells at a lower dosage of 10 µM, with BA1 showing a drop of 19.6%, BA2 18.7%, BA3 13.6%, and BA4 32.3%. When administered at a consistent concentration of 10 µM, BI exhibited a reduction in cell viability of just 25.0%. At increased doses of 50 and 75 µM, BA2, BA3, and BA4 showed cell viability within the range of −0.4% and 5.8% [49].

In the current study, the MTT assay was employed to evaluate the cytotoxic effects of the aforementioned derivatives, as well as the known compounds BA3, BA4, and the parent compound BI. The objective was to quantitatively measure the variations in cell viability resulting from the chemical modifications against B164A5 melanoma cells. The experimental analysis revealed that all of the examined concentrations, namely 1, 10, 25, 50, and 75 µM, exhibited a substantial reduction in cell viability in a way that was dependent on the dosage, as compared to the control group. Compound BA3 exhibited the most pronounced cytotoxic effects on the B164A5 cell line, resulting in a reduction in growth ranging from −1.5% to 8.1%. At the lowest concentration of 1µM, the cell viability dropped to 8.1% and 15.9% for BA3 and BA2 compared to 31.3% for BI. The maximum inhibitory effect on cell viability was observed at the highest concentration examined (75 µM) when the growth percentages reached the values of 5.7% for BA1, −0.4% for BA2, −1.5% for BA3, 15% for BA4, and 9.6% for BI.

The results of this experiment demonstrated that the introduction of an indole skeleton at the C2 position of BI led to a significant increase in cytotoxic activity. BA4 was 1.2 times more active than the parent triterpenoid with IC_50_ of 17.62 μM. Moreover, the augmentation of cytotoxicity was seen upon the conjugation of the carboxylic group of compound BA4 with an amino acid residue. BA3 and BA2 containing glycine and glycylglycine residues at C28 position exhibited a nearly 2.20-fold higher inhibitory activity (IC_50_ = 8.11 μM, IC_50_ = 9.15 μM) compared to BA4 (IC_50_ = 17.62 μM). At elevated doses, namely at 50 μM and 75 μM, the aforementioned compounds have shown negative cell viability. This observation may be attributed to the phenomenon whereby deceased cells lose their capacity to convert tetrazolium salts into colorful formazan products. Therefore, it has been shown that higher concentrations of BA3 and BA2 result in the induction of apoptosis, as validated via nuclear staining with Hoechst 33342. In relation to the inhibitory effects of betulinic acid on the B164A5 cell line, our findings exhibit a level of similarity to the outcomes reported by previous authors. Farcas et al. observed a reduction in cell viability of murine melanoma cells B164A5 by 40% following 48 h of exposure to BI (25 µM) [67]. In contrast, the current investigation observed that BI, when administered at an equivalent dose, resulted in a reduction in cell viability by 71% over a 72 h exposure period. Soica et al. carried out research on the cytotoxic effects of BI on B164A5 cells. After 72 h of exposure, BI exhibited cytotoxic action, resulting in 50% cell viability at a concentration of 10 mM [68]. Khusnutdinova et al. examined the possible cytotoxic effects of 2,3-indolo-betulinic acid at a concentration of 100 µM on several melanoma cell lines, including MDA-MB-435, MALME-3M, LOX IMVI, MALME-M14, SK-MEL-2, SK-MEL-28, SK-MEL-5, UACC-257, and UACC-62. The Lox IMVI melanoma cells had the greatest degree of reactivity to the derivative under investigation during a 48 h period of stimulation, resulting in a viability percentage of 44%. Following closely after were the SK-MEL-2 cells, which exhibited a viability rate of 57% [69]. Jeong et al. successfully synthesized conjugates of C-28 amino acids, resulting in enhanced targeted toxicity against human melanoma (MEL-2) and improved water solubility of BI. The enhancement of cytotoxicity towards MEL-2 (IC_50_ = from 10.2 to 4.2 µg/mL) was observed upon conversion of methyl ester of glycine conjugates to their corresponding free acid conjugates. The researchers came to the conclusion that the glycine-free acid exhibited remarkable solubility in water while maintaining the particular cytotoxicity of the original compound, betulinic acid [70]. The safety characteristics of the test compounds were evaluated by conducting a cell viability assessment on a healthy cell line: human keratinocytes-HaCat. Our research revealed that when HaCat cells were exposed to a 72 h period, there was a decline in cell viability that was dependent on the dosage administered (Figure 3). Our results suggest that betulinic acid derivatives at concentrations up to 10 µM were linked to a slight reduction in the percentage of viable HaCat cells, with viability rates ranging from 79% to 98%. The maximum concentration of BA1, BA2, and BA3, which was 75 M, resulted in a relatively low percentage of viable cells for HaCat, approximately 70%. Out of the four derivatives of betulinic acid, BA4 demonstrated the most secure characteristics on HaCaT, exhibiting a cell viability rate of 80.3% at a concentration of 75 µM. The cell viability data validate that BA1–BA4 demonstrate selective cytotoxicity towards human melanoma cells (A375) [49] and murine melanoma cells (B164A5), while their toxicity towards normal cells can be defined as low to moderate. With respect to the influence of BI on HaCaT cells, our findings suggest that cell viability was diminished by approximately 20% at concentrations exceeding 10 µM. The findings are consistent with the data that have been published by other researchers. Coricovac et al. have shown that concentrations over 10 μM of BI resulted in a 20% decrease in cell viability and caused significant changes in the morphology of HaCaT cells, as well as modifications in their nuclei [31]. The group of Wróblewska-Łuczka noticed a notable decrease in the viability of HaCaT cells after 72 h of exposure to BI at doses ranging from 16 to 40 µM. The cell viability rates ranged from 60% to 90% [71].

The LDH assessment is used to quantify the release of lactate dehydrogenase, a stable cytosolic enzyme, into the surrounding culture media. This measurement serves as a signal of irreversible damage to the cell membrane and subsequent cell death [72]. The present findings support the results previously published by our research team, which demonstrated that the compounds BA2, BA3, and BA4 exhibited the highest cytotoxic activity against human melanoma A375 cells at a concentration of 25 µM. Specifically, BA2, BA3, and BA4 induced a cytotoxic effect of 39.13%, 30.4%, and 43.36%, respectively, while BI resulted in a cytotoxic effect of 21.2%. At higher dosages (75 µM), there was a small reduction in the cytotoxic rate seen for BA2 (23.69%), BA3 (20.54%), and BA4 (32.8%). The aforementioned inclination was previously documented in studies involving the A375 human melanoma cell line, whereby chemicals capable of inducing cell cycle arrest were examined. Considering the observation that cells have lost their ability to undergo proliferation, it can be inferred that the production of LDH from these cells would be relatively limited. Consequently, this decrease in LDH production will result in a reduction in the cytotoxic effect exerted by the sample [48]. In the present investigation, it was observed that during a 72 h incubation period, the application of all examined substances at a concentration of 25 µM or above resulted in a significant release of LDH in murine melanoma cells (B164A5) (Figure 4). At a dose of 75 µM, the two novel compounds (BA1 and BA2) demonstrated significant cytotoxic activity—44.3% and 62.14%, respectively. Among the compounds that were analyzed, it was observed that BA3 (at a concentration of 75 µM) had the highest cytotoxic impact against B164A5 cells, resulting in a cytotoxic rate of 80.68%. This value was compared to the cytotoxic effect of the reference chemical BI, which showed a cell cytotoxic rate of 52.7%. The observed cytotoxicity of the analyzed compounds aligns with the metabolic activity of B164A5 as determined by the MTT assay. The compounds BA2 and BA3 demonstrated a significant reduction in cell viability, with IC_50_ values of 9.15 μM and 8.11 μM, respectively. Additionally, the LDH assay provided further evidence of the cytotoxic effects of these compounds.

The Neutral Red Uptake (NR) assay is a colorimetric technique used to evaluate cytotoxicity in an in vitro setting. The technique relies on the capacity of living B164A5 cells to incorporate neutral red dye into their lysosomes. The experimental procedure is dependent on the capacity of viable cells to assimilate and attach neutral red, a mildly cationic dye, within their lysosomes. Therefore, the expression of cytotoxicity is observed as a reduction in the uptake of neutral red, which is dependent on the concentration, following exposure to the xenobiotic being studied [73]. The results demonstrate that there is a dose–response relationship identified for all chemicals (Figure 5). It is worth mentioning that BA1 had a considerable cytotoxic effect at a concentration of 75 µM, resulting in a cytotoxic rate of 77.5%. Similarly, BA2 and BA3 demonstrated cytotoxicity rates of 69.9% and 64.2%, respectively, when compared to the original chemical BI, which exhibited a cytotoxicity rate of 63.0%. The results obtained from the NR assessment support the data obtained from the MTT assay, where the application of BA1, BA2, and BA3 at a concentration of 75 µM significantly reduced cell viability (Figure 2).

The impact of 2,3-indolo-betulinic acid derivatives and BI on cellular proliferation and migration was evaluated by the use of the scratch assessment, a technique akin to wound healing. In our prior study using the human melanoma cell line A375, all the assessed chemicals decreased the melanoma cells’ capacity to migrate in a dose-dependent fashion when compared to the control cells. The tested substances severely impeded the migration of cells at higher concentrations (25 µM and 50 µM), resulting in a decrease of 73.2% to 2.1%. The application of a 25 µM concentration of BI to cells demonstrated a considerable inhibition of cell migration, leading to scratch closure rates of 17.5%. The two novel substances, BA1 and BA2, have shown significant inhibitory effects on cell migration, as evidenced by wound healing rates of 35% and 30%, respectively, when administered at a concentration of 50 µM. The compound BA4 exhibited the least significant enhancement in cell migration, which was strongly correlated with its unfavorable cytotoxic characteristics [48]. On the other hand, in the current investigation, the B164A5 control cells demonstrated migratory behavior by completely covering the wound area within 24 h. In contrast, cells treated with the compounds under investigation showed a reduction in the migratory process of the cells (Figure 6). The findings of the scratch assessment align with the antiproliferative outcomes obtained from the MTT assay. The anti-migratory effect of BA2 and BA3 appeared to be the most pronounced when tested at a concentration of 50 µm, resulting in scratch closure rates of 10.0% and 21.3%, respectively. These results demonstrate that the rate of cell migration was closely related to the cytotoxic profile of the compounds.

One further morphological characteristic that reflects the cytotoxicity of a substance is the observation of nuclear alterations, which serve as indicators of the existence of apoptotic or necrotic cells. In order to determine the specific mechanism of cell death triggered by BI, BA1, BA2, BA3, and BA4 in B164A5 cells, the nuclei were subjected to staining with Hoechst 33342 dye for validation purposes. Concentrations of 25, 50, and 75 μM were used, since these values were chosen based on the outcomes of cell viability assessments. Therefore, as illustrated in Figure 7, it can be observed that the cells treated with DMSO did not exhibit any indications of apoptosis or necrosis. On the other hand, the BA1 sample induced apoptotic characteristics only at a concentration of 75 μM, while the cells treated with BA2 displayed distinct signs of apoptosis, such as chromatin condensation, at both concentrations of 50 and 75 μM. However, it is worth noting that the BA3 sample resulted in the most significant nuclear alterations, with the necrotic process being apparent at a dosage of 25 μM. On the other hand, necrosis was evident in cells exposed to BA4 and BI only at a concentration of 50 μM. The observed results demonstrate a clear association with the MTT test, as BA2 concentrations of 50 and 75 µM showed a decrease in cell viability rates (0.41% and −0.43%), indicating a potential link to apoptotic processes. In a comparable fashion, the compound BA3, which has shown high cytotoxicity, displayed a decline in cell viability at concentrations as low as 25 µM. This decline in viability was found to be associated with the observed necrotic process, as evidenced by the use of nuclear staining assay.

## 4. Materials and Methods

### 4.1. Cell Culture

The B164A5 cell line was obtained from Sigma Aldrich (ECACC) and HaCaT cell line was obtained from ATCC (LGC Standards GmbH, Wesel, Germany). The cells in question were cultivated in a comprehensive growth medium, which consists of DMEM (Dulbecco’s Modified Eagle’s Medium) enriched with 10% FCS (Foetal Calf Serum), 1% Penicillin/Streptomycin combination (Pen/Strep, 10,000 IU/mL), and 2% HEPES (4-(2-hydroxyethyl)-1-piperazineethanesulfonic acid). The cells were cultivated by incubation at a temperature of 37 °C in an environment containing 5% CO_2_. At a confluence level ranging from 70% to 80% occurring every two or three days, the cells were subjected to passaging using a solution consisting of 0.25% Trypsin and 1 mM EDTA. This was followed by centrifugation at 1200 rpm for 5 min, and afterward, the cells were replated in T75 culture flasks. The subcultivation rate of 1:10 was selected to promote maximum cell proliferation.

### 4.2. Cellular Viability

The MTT assay was used to determine the effect of 2,3-indolo-betulinic acid derivatives and betulinic acid on the viability of HaCaT human keratinocyte cell line and B164A5 murine melanoma cells. The methodology was executed in accordance with the previously outlined procedure [48,74]. Briefly, 1 × 10^4^ cells per well were seeded onto 96-well culture plates and incubated until 70–80% confluence was achieved. Subsequently, the cells were subjected to stimulation for a duration of 72 h using five distinct concentrations (1, 10, 25, 50, and 75 µM) of the test samples. Following a 72 h incubation period, a 10 μL amount of a 5 mg/mL MTT solution obtained from the MTT kit (Sigma-Aldrich, St. Louis, MO, USA) was introduced into each well and subjected to an additional 3 h incubation. The formazan crystals that had been yielded were dissolved by the addition of 100 μL per well of the lysis solution that was included in the MTT kit. B164A5 cells and HaCaT cells were used as a control, and they were only treated with culture medium for 72 h. The spectrophotometric analysis of absorbance was performed at a wavelength of 570 nm using a microplate reader (BioRad, Hercules, CA, USA, xMark Microplate Spectrophotometer).

### 4.3. Evaluation of the Cytotoxic Potential by Lactate Dehydrogenase (LDH) Assay

The LDH technique is used to evaluate the extracellular release of internal lactate dehydrogenase subsequent to the disruption of the cellular membrane. Consequently, this approach offers insights into occurrences resembling necrosis [75].

The present investigation used the LDH test to assess the cytotoxicity and necrosis-related events that transpired after the exposure of B164A5 murine melanoma cells to the test samples for a duration of 72 h.

The experimental procedure was conducted in accordance with the instructions outlined in the pierce LDH cytotoxicity assay kit, which was supplied by the manufacturer (Thermo Fisher Scientific, Boston, MA, USA). A comprehensive description of this protocol can be found in our previous publications [76,77].

### 4.4. Neutral Red (NR) Assay

In addition to undertaking the MTT proliferation assessment, the NR technique was used to measure the cytotoxicity rate of the substances being studied. The underlying premise of this methodology depends upon the measurement of live cells using the NR chromophore, which effectively stains the lysosome of living cells. The incorporation of the dye is achieved by active transport, hence rendering non-living cells incapable of assimilating the NR reagent. Consequently, live cells have the capability to secrete the NR dye when subjected to acidic conditions. Moreover, the intensity of the dye may be directly associated with the quantity of living cells, thereby enabling the assessment of drug-induced cytotoxicity.

In summary, the experimental procedure included subjecting the cells to five different doses (1, 10, 25, 50, and 75 µM) of test samples for a duration of 72 h. Subsequently, a volume of 10 μL of a 0.33% NR solution was introduced into each well, followed by an incubation period of at least 3 h. Following the completion of the incubation time, the medium was extracted, and the cells were promptly rinsed with a 50 μL solution of NR assay fixative. Subsequently, a solubilization step was carried out using a 100 μL solution of NR assay solubilization solution per well.

### 4.5. Anti-Migratory Potential—Scratch Assay Method

To evaluate the invasion capability of B164A5 cells after stimulation with the tested compounds (1, 10, 25, and 50 µM), a scratch-based experiment was conducted. In summary, a quantity of 2 × 10^5^ cells/well were evenly distributed onto 12-well culture plates until a complete and cohesive layer of cells was achieved. Subsequently, a sterile pipette tip was used to create a void in the center of each well. The cells and cellular debris that were detached subsequent to the process were carefully rinsed using phosphate buffer saline (PBS). Additionally, the cells were subjected to stimulation with the tested compounds. Photographs of the scratched region were captured at two time points: 0 h and 24 h. The images were obtained using an inverted microscope (Olympus IX73, Olympus, Tokyo, Japan) with a DP74 camera (Olympus, Tokyo, Japan) at a magnification of 10×. The cellSense Dimension program was used for the purpose of analyzing cell growth.

The scratch closure rate was calculated using the formula [78]:

Scratch closure rate = [(A_t0_ − A_t_)/A_t_] × 100,

where A_t0_ represents the initial scratch area at time 0 h, and A_t_ represents the scratch area after 24 h.

### 4.6. Hoechst Staining

To assess the nuclear-level effects of 2,3-indolo-betulinic acid derivatives and betulinic acid (at concentrations of 1, 10, 25, 50, and 75 µM) on tumor (B164A5) cells after a 24 h treatment, the Hoechst 33342 staining test was conducted. The procedure was implemented in accordance with the manufacturer’s recommendations. The experimental procedure involved the following steps: (i) the seeding of cells at a density of 1 × 10^5^ cells/1.5 mL medium per well in a 12-well plate; (ii) the treatment of cells with varying concentrations of tested compounds in DMSO (1, 10, 25, 50, and 75 µM) for a duration of 24 h; (iii) the removal of the previous media, which contained the test compound, after the treatment period, followed by the addition of 0.5 mL of the staining solution (diluted at a ratio of 1:2000 in PBS) to each well; (iv) the incubation of the cells for 10 min at room temperature, while ensuring protection from light; (v) the removal of the staining solution and subsequent washing of the cells three times with PBS; and (vi) the capturing of images under UV irradiation by employing the Olympus IX73 (Olympus, Tokyo, Japan) inverted microscope with an integrated DP74 digital camera (Olympus, Tokyo, Japan). A positive control was used to induce apoptosis, using a concentration of 5 µM staurosporine for a duration of 3 h at a temperature of 37 °C. Similarly, for necrosis induction, a concentration of 0.5% Triton X-100 was utilized, with an incubation period of 30 min at 37 °C.

### 4.7. Statistical Analysis

The process of collecting data and conducting statistical analysis was carried out using GraphPad Prism 9.3.1, a software developed by GraphPad Software in San Diego, CA, USA. The data are presented as the average of three distinct experiments, together with the standard deviation (SD). To evaluate the statistical differences, a one-way ANOVA and Dunnett’s multiple comparisons post-test were employed (* *p* ≤ 0.05; ** *p* ≤ 0.01; *** *p* ≤ 0.001; **** *p* ≤ 0.0001).

## 5. Conclusions

We have successfully demonstrated that our recently designed semi-synthetic betulinic acid derivatives *N*-(2,3-indolo-betulinoyl)diglycylglycine (BA1) and *N*-(2,3-indolo-betulinoyl)glycylglycine (BA2) possess a significant biological activity profile on B164A5 cell line. The obtained data were validated against previously known *N*-(2,3-indolo-betulinoyl)glycine (BA3), 2,3-indolo-betulinic acid (BA4) and betulinic acid (BI) as natural product standard. The cytotoxicity assessment on the B164A5 cells showed that all compounds significantly reduced cell viability, with BA3 and BA2 showing the highest cytotoxicity, reflected in low IC_50_ values. The evaluation of the safety profile on human keratinocytes (HaCaT cells) revealed that doses of the derivatives up to 10 µM exhibited a modest decrease in cell viability; however, a concentration of 75 µM led to a lowered proportion of viable cells. LDH leakage, indicating cell membrane disruption, was concentration-dependent and pronounced in BA2 and BA3. Similar cytotoxic patterns were observed in NR assay. Wound healing assay demonstrated concentration-dependent inhibitory effects on migratory activity, with BA2 and BA3 exhibiting substantial inhibition. Additionally, the compounds induced apoptotic characteristics and altered cell nuclei morphology, further supporting their cytotoxic effects. Overall, the results suggest that the 2,3-indolo-betulinic acid derivatives, particularly BA2 and BA3, have promising potential as cytotoxic agents against B164A5 cells and deserve further investigation as potential anticancer therapeutics.

## Figures and Tables

**Table 1:** IC_50_ (µM) values of BA1-BA4 and BI on B164A5 murine melanoma cells by MTT assay.

Compound	IC_50_ (μM)
BI	21.14 ± 0.08
BA1	10.34 ± 0.06
BA2	9.15 ± 0.05
BA3	8.11 ± 0.13
BA4	17.62 ± 0.11

## Data Availability

Data are contained within the article and Supplementary Materials.

## Supplementary Materials

The following supporting information can be downloaded at https://www.mdpi.com/article/10.3390/plants13010036/s1: Figure S1: Anti-migratory activity of BA1 and BA2; Figure S2: Anti-migratory activity of BA3 and BA4; Figure S3: Anti-migratory activity of BI.

## References

[1] Siddiqui A.J., Jahan S., Singh R., Saxena J., Ashraf S.A., Khan A., Choudhary R.K., Balakrishnan S., Badraoui R., Bardakci F. (2022). Review Article Plants in Anticancer Drug Discovery: From Molecular Mechanism to Chemoprevention. BioMed Res. Int..

[2] Cragg G.M., Newman D.J., Snader K.M. (2000). The Influence of Natural Products upon Drug Discovery. Nat. Prod. Rep..

[3] Butler M.S. (2004). The Role of Natural Product Chemistry in Drug Discovery. J. Nat. Prod..

[4] Bhusnure O.G., Shinde M.C., Vijayendra S.S.M., Gholve S.B., Giram P.S., Birajdar M.J. (2019). Phytopharmaceuticals: An Emerging Platform for Innovation and Development of New Drugs from Botanicals. J. Drug Deliv. Ther..

[5] Atanasov A.G., Waltenberger B., Pferschy-Wenzig E.M., Linder T., Wawrosch C., Uhrin P., Temml V., Wang L., Schwaiger S., Heiss E.H. (2015). Discovery and Resupply of Pharmacologically Active Plant-Derived Natural Products: A Review. Biotechnol. Adv..

[6] Rates S.M.K. (2001). Plants as Source of Drugs. Toxicon.

[7] Izzo A.A., Hoon-Kim S., Radhakrishnan R., Williamson E.M., Fürst R., Zündorf I., Bhusnure O.G., Shinde M.C., Vijayendra S.S.M., Gholve S.B. (2020). A Critical Approach to Evaluating Clinical Efficacy, Adverse Events and Drug Interactions of Herbal Remedies. Phytother. Res..

[8] Newman D.J., Cragg G.M. (2020). Natural Products as Sources of New Drugs over the Nearly Four Decades from 01/1981 to 09/2019. J. Nat. Prod..

[9] Ignatenko V.A., Han Y., Tochtrop G.P. (2013). Molecular Library Synthesis Using Complex Substrates: Expanding the Framework of Triterpenoids. J. Org. Chem..

[10] Blundell R., Azzopardi J., Briffa J., Rasul A., Vargas-de la Cruz C., Shah M.A. (2020). Analysis of Pentaterpenoids. Recent Advances in Natural Products Analysis.

[11] Haque A., Brazeau D., Amin A.R. (2021). Perspectives on Natural Compounds in Chemoprevention and Treatment of Cancer: An Update with New Promising Compounds. Eur. J. Cancer.

[12] Hoenke S., Heise N.V., Kahnt M., Deigner H.P., Csuk R. (2020). Betulinic Acid Derived Amides Are Highly Cytotoxic, Apoptotic and Selective. Eur. J. Med. Chem..

[13] Lombrea A., Scurtu A.D., Avram S., Pavel I.Z., Turks M., Lugiņina J., Peipiņš U., Dehelean C.A., Soica C., Danciu C. (2021). Anticancer Potential of Betulonic Acid Derivatives. Int. J. Mol. Sci..

[14] Pavel I.Z., Pârvu A.E., Dehelean C.A., Vlase L., Csuk R., Muntean D.M. (2017). Assessment of the Antioxidant Effect of a Maslinic Acid Derivative in an Experimental Model of Acute Inflammation. Farmacia.

[15] Saxena B.B., Zhu L., Hao M., Kisilis E., Katdare M., Oktem O., Bomshteyn A., Rathnam P. (2006). Boc-Lysinated-Betulonic Acid: A Potent, Anti-Prostate Cancer Agent. Bioorg. Med. Chem..

[16] Kozubek M., Hoenke S., Deigner H.P., Csuk R. (2022). Betulinic Acid and Glycyrrhetinic Acid Derived Piperazinyl Spacered Rhodamine B Conjugates Are Highly Cytotoxic and Necrotic. Results Chem..

[17] Khusnutdinova E.F., Petrova A.V., Thu H.N.T., Tu A.L.T., Thanh T.N., Thi C.B., Babkov D.A., Kazakova O.B. (2019). Structural Modifications of 2,3-Indolobetulinic Acid: Design and Synthesis of Highly Potent α-Glucosidase Inhibitors. Bioorg. Chem..

[18] Isah M.B., Ibrahim M.A., Mohammed A., Aliyu A.B., Masola B., Coetzer T.H.T. (2016). A Systematic Review of Pentacyclic Triterpenes and Their Derivatives as Chemotherapeutic Agents against Tropical Parasitic Diseases. Parasitology.

[19] Jäger S., Trojan H., Kopp T., Laszczyk M.N., Scheffler A. (2009). Pentacyclic Triterpene Distribution in Various Plants—Rich Sources for a New Group of Multi-Potent Plant Extracts. Molecules.

[20] Jonnalagadda S.C., Suman P., Morgan D.C., Seay J.N. (2017). Recent Developments on the Synthesis and Applications of Betulin and Betulinic Acid Derivatives as Therapeutic Agents.

[21] Pavlova N.I., Savinova O.V., Nikolaeva S.N., Boreko E.I., Flekhter O.B. (2003). Antiviral Activity of Betulin, Betulinic and Betulonic Acids against Some Enveloped and Non-Enveloped Viruses. Fitoterapia.

[22] Kazakova O.B., Giniyatullina G.V., Mustafin A.G., Babkov D.A., Sokolova E.V., Spasov A.A. (2020). Evaluation of Cytotoxicity and A-glucosidase Inhibitory Activity of Amide and Polyamino-derivatives of Lupane Triterpenoids. Molecules.

[23] Tsepaeva O.V., Nemtarev A.V., Salikhova T.I., Abdullin T.I., Grigor’eva L.R., Khozyainova S.A., Mironov V.F. (2019). Synthesis, Anticancer, and Antibacterial Activity of Betulinic and Betulonic Acid C-28-Triphenylphosphonium Conjugates with Variable Alkyl Linker Length. Anticancer Agents Med. Chem..

[24] Szuster-Ciesielska A., Plewka K., Kandefer-Szerszeń M. (2011). Betulin, Betulinic Acid and Butein Are Inhibitors of Acetaldehyde-Induced Activation of Liver Stellate Cells. Pharmacol. Rep..

[25] Regueiro-Ren A., Liu Z., Chen Y., Sin N., Sit S.-Y., Swidorski J.J., Chen J., Venables B.L., Zhu J., Nowicka-Sans B. (2016). Discovery of BMS-955176, a Second Generation HIV-1 Maturation Inhibitor with Broad Spectrum Antiviral Activity. ACS Med. Chem. Lett..

[26] Sorokina I.V., Tolstikova T.G., Zhukova N.A., Petrenko N.I., Schults E.E., Uzenkova N.V., Grek O.R., Pozdnyakova S.V., Tolstikov G.A. (2004). Betulonic Acid and Derivatives, a New Group of Agents Reducing Side Effects of Cytostatics. Dokl. Biol. Sci..

[27] Sousa J.L.C., Freire C.S.R., Silvestre A.J.D., Silva A.M.S. (2019). Recent Developments in the Functionalization of Betulinic Acid and Its Natural Analogues: A Route to New Bioactive Compounds. Molecules.

[28] Lugiņina J., Linden M., Bazulis M., Kumpiņš V., Mishnev A., Popov S.A., Golubeva T.S., Waldvogel S.R., Shults E.E., Turks M. (2021). Electrosynthesis of Stable Betulin-Derived Nitrile Oxides and Their Application in Synthesis of Cytostatic Lupane-Type Triterpenoid-Isoxazole Conjugates. Eur. J. Org. Chem..

[29] Mierina I., Vilskersts R., Turks M. (2020). Delivery Systems for Birch-Bark Triterpenoids and Their Derivatives in Anticancer Research. Curr. Med. Chem..

[30] Pisha E., Chai H., Lee I.-S., Chagwedera T.E., Farnsworth N.R., Cordell G.A., Beecher C.W.W., Fong H.H.S., Kinghorn A.D., Brown D.M. (1995). Discovery of Betulinic Acid as a Selective Inhibitor of Human Melanoma That Functions by Induction of Apoptosis. Nat. Med..

[31] Coricovac D., Dehelean C.A., Pinzaru I., Mioc A., Aburel O.-M., Macasoi I., Draghici G.A., Petean C., Soica C., Boruga M. (2021). Assessment of Betulinic Acid Cytotoxicity and Mitochondrial Metabolism Impairment in a Human Melanoma Cell Line. Int. J. Mol. Sci..

[32] Takada Y., Aggarwal B.B. (2003). Betulinic Acid Suppresses Carcinogen-Induced NF-κB Activation through Inhibition of IκBα Kinase and P65 Phosphorylation: Abrogation of Cyclooxygenase-2 and Matrix Metalloprotease-9. J. Immunol..

[33] Melzig M.F., Bormann H. (1998). Betulinic Acid Inhibits Aminopeptidase N Activity. Planta Med..

[34] Kwon H.J., Shim J.S., Kim J.H., Cho H.Y., Yum Y.N., Kim S.H., Yu J. (2002). Betulinic Acid Inhibits Growth Factor-induced in Vitro Angiogenesis via the Modulation of Mitochondrial Function in Endothelial Cells. Jpn. J. Cancer Res..

[35] Zhong Y., Liang N., Yang L.I.U., Cheng M.-S. (2021). Recent Progress on Betulinic Acid and Its Derivatives as Antitumor Agents: A Mini Review. Chin. J. Nat. Med..

[36] Nistor G., Mioc A., Mioc M., Balan-Porcarasu M., Ghiulai R., Racoviceanu R., Avram Ș., Prodea A., Semenescu A., Milan A. (2022). Novel Semisynthetic Betulinic Acid−Triazole Hybrids with In Vitro Antiproliferative Potential. Processes.

[37] Nistor G., Mioc M., Mioc A., Balan-Porcarasu M., Racoviceanu R., Prodea A., Milan A., Ghiulai R., Semenescu A., Dehelean C. (2022). The C30-Modulation of Betulinic Acid Using 1,2,4-Triazole: A Promising Strategy for Increasing Its Antimelanoma Cytotoxic Potential. Molecules.

[38] Kim D.S.H.L., Pezzuto J.M., Pisha E. (1998). Synthesis of Betulinic Acid Derivatives with Activity against Human Melanoma. Bioorg. Med. Chem. Lett..

[39] Kumar V., Rani N., Aggarwal P., Sanna V.K., Singh A.T., Jaggi M., Joshi N., Sharma P.K., Irchhaiya R., Burman A.C. (2008). Synthesis and Cytotoxic Activity of Heterocyclic Ring-Substituted Betulinic Acid Derivatives. Bioorg. Med. Chem. Lett..

[40] Yang S.-J., Liu M.-C., Xiang H.-M., Zhao Q., Xue W., Yang S. (2015). Synthesis and in Vitro Antitumor Evaluation of Betulin Acid Ester Derivatives as Novel Apoptosis Inducers. Eur. J. Med. Chem..

[41] Ferlay J., Colombet M., Soerjomataram I., Parkin D.M., Piñeros M., Znaor A., Bray F. (2021). Cancer Statistics for the Year 2020: An Overview. Int. J. Cancer.

[42] Tilaoui M., Ait Mouse H., Zyad A. (2021). Update and New Insights on Future Cancer Drug Candidates from Plant-Based Alkaloids. Front. Pharmacol..

[43] Apalla Z., Lallas A., Sotiriou E., Lazaridou E., Ioannides D. (2017). Epidemiological Trends in Skin Cancer What Does the Future Hold. Dermatol. Pract. Concept..

[44] Strashilov S., Yordanov A. (2021). Aetiology and Pathogenesis of Cutaneous Melanoma: Current Concepts and Advances. Int. J. Mol. Sci..

[45] Becquart O., Oriano B., Dalle S., Mortier L., Leccia M.T., Dutriaux C., Dalac S., Montaudié H., De Quatrebarbes J., Brunet-Possenti F. (2021). Tolerance and Effectiveness of Targeted Therapies in Aged Patients with Metastatic Melanoma. Cancers.

[46] Wróbel S., Przybyło M., Stępień E. (2019). The Clinical Trial Landscape for Melanoma Therapies. J. Clin. Med..

[47] Nistor M., Rugina D., Diaconeasa Z., Socaciu C., Socaciu M.A. (2023). Pentacyclic Triterpenoid Phytochemicals with Anticancer Activity: Updated Studies on Mechanisms and Targeted Delivery. Int. J. Mol. Sci..

[48] Lombrea A., Semenescu A.D., Magyari-Pavel I.Z., Turks M., Lugiņina J., Peipiņš U., Muntean D., Dehelean C.A., Dinu S., Danciu C. (2023). Comparison of In Vitro Antimelanoma and Antimicrobial Activity of 2,3-Indolo-Betulinic Acid and Its Glycine Conjugates. Plants.

[49] Wang J.X., Fukunaga-Kalabis M., Herlyn M. (2016). Crosstalk in Skin: Melanocytes, Keratinocytes, Stem Cells, and Melanoma. J. Cell Commun. Signal..

[50] Wu Y., Yuan Z., Rao Y. (2023). Current Advances in the Biotechnological Synthesis of Betulinic Acid: New Findings and Practical Applications. Syst. Microbiol. Biomanuf..

[51] Jiang W., Li X., Dong S., Zhou W. (2021). Betulinic Acid in the Treatment of Tumour Diseases: Application and Research Progress. Biomed. Pharmacother..

[52] Grymel M., Zawojak M., Adamek J. (2019). Triphenylphosphonium Analogues of Betulin and Betulinic Acid with Biological Activity: A Comprehensive Review. J. Nat. Prod..

[53] Drag-Zalesinska M., Kulbacka J., Saczko J., Wysocka T., Zabel M., Surowiak P., Drag M. (2009). Esters of Betulin and Betulinic Acid with Amino Acids Have Improved Water Solubility and Are Selectively Cytotoxic toward Cancer Cells. Bioorg. Med. Chem. Lett..

[54] Amiri S., Dastghaib S., Ahmadi M., Mehrbod P., Khadem F., Behrouj H., Aghanoori M.R., Machaj F., Ghamsari M., Rosik J. (2020). Betulin and Its Derivatives as Novel Compounds with Different Pharmacological Effects. Biotechnol. Adv..

[55] Govdi A.I., Sokolova N.V., Sorokina I.V., Baev D.S., Tolstikova T.G., Mamatyuk V.I., Fadeev D.S., Vasilevsky S.F., Nenajdenko V.G. (2015). Synthesis of New Betulinic Acid–Peptide Conjugates and in Vivo and in Silico Studies of the Influence of Peptide Moieties on the Triterpenoid Core Activity. Med. Chem. Commun..

[56] Popov S.A., Semenova M.D., Baev D.S., Sorokina I.V., Zhukova N.A., Frolova T.S., Tolstikova T.G., Shults E.E., Turks M. (2019). Lupane-Type Conjugates with Aminoacids, 1, 3, 4-Oxadiazole and 1, 2, 5-Oxadiazole-2-Oxide Derivatives: Synthesis, Anti-Inflammatory Activity and in Silico Evaluation of Target Affinity. Steroids.

[57] Popov S.A., Semenova M.D., Baev D.S., Frolova T.S., Shults E.E., Wang C., Turks M. (2020). Synthesis of Cytotoxic Urs-12-Ene-and 28-Norurs-12-Ene-Type Conjugates with Amino-and Mercapto-1, 3, 4-Oxadiazoles and Mercapto-1, 2, 4-Triazoles. Steroids.

[58] Popov S.A., Wang C., Qi Z., Shults E.E., Turks M. (2021). Synthesis of Water-Soluble Ester-Linked Ursolic Acid–Gallic Acid Hybrids with Various Hydrolytic Stabilities. Synth. Commun..

[59] Semenova M.D., Popov S.A., Golubeva T.S., Baev D.S., Shults E.E., Turks M. (2021). Synthesis and Cytotoxicity of Sulfanyl, Sulfinyl and Sulfonyl Group Containing Ursane Conjugates with 1, 3, 4-Oxadiazoles and 1, 2, 4-Triazoles. ChemistrySelect.

[60] Khlebnicova T.S., Piven Y.A., Baranovsky A.V., Lakhvich F.A., Shishkina S.V., Zicāne D., Tetere Z., Rāviņa I., Kumpiņš V., Rijkure I. (2017). Synthesis of Novel Lupane Triterpenoid-Indazolone Hybrids with Oxime Ester Linkage. Steroids.

[61] Luo M.L., Zhao Q., He X.H., Xie X., Zhu H.P., You F.M., Peng C., Zhan G., Huang W. (2023). Research Progress of Indole-Fused Derivatives as Allosteric Modulators: Opportunities for Drug Development. Biomed. Pharmacother..

[62] Luo M.L., Huang W., Zhu H.P., Peng C., Zhao Q., Han B. (2022). Advances in Indole-Containing Alkaloids as Potential Anticancer Agents by Regulating Autophagy. Biomed. Pharmacother..

[63] Han B., He X.H., Liu Y.Q., He G., Peng C., Li J.L. (2021). Asymmetric Organocatalysis: An Enabling Technology for Medicinal Chemistry. Chem. Soc. Rev..

[64] Qin R., Zhao Q., Han B., Zhu H.P., Peng C., Zhan G., Huang W. (2022). Indole-Based Small Molecules as Potential Therapeutic Agents for the Treatment of Fibrosis. Front. Pharmacol..

[65] Baillie G.L., Horswill J.G., Anavi-Goffer S., Reggio P.H., Bolognini D., Abood M.E., McAllister S., Strange P.G., Stephens G.J., Pertwee R.G. (2013). CB_1_ Receptor Allosteric Modulators Display Both Agonist and Signaling Pathway Specificity. Mol. Pharmacol..

[66] Shao Z., Yan W., Chapman K., Ramesh K., Ferrell A.J., Yin J., Wang X., Xu Q., Rosenbaum D.M. (2019). Structure of an Allosteric Modulator Bound to the CB1 Cannabinoid Receptor. Nat. Chem. Biol..

[67] Farcas C.G., Moaca E.A., Dragoi R., Vaduva D.B., Marcovici I., Mihali C.V., Loghin F. (2019). Preliminary Results of Betulinic Acid-Loaded Magnetoliposomes—A Potential Approach to Increase Therapeutic Efficacy in Melanoma. Rev. Chim..

[68] Soica C., Danciu C., Savoiu-Balint G., Borcan F., Ambrus R., Zupko I., Bojin F., Coricovac D., Ciurlea S., Avram S. (2014). Betulinic Acid in Complex with a Gamma-Cyclodextrin Derivative Decreases Proliferation and in Vivo Tumor Development of Non-Metastatic and Metastatic B164A5 Cells. Int. J. Mol. Sci..

[69] Khusnutdinova E.F., Petrova A.V., Apryshko G.N., Kukovinets O.S., Kazakova O.B. (2018). Synthesis and Cytotoxicity of Indole Derivatives of Betulin, Erythrodiol, and Uvaol. Russ. J. Bioorg. Chem..

[70] Jeong H.J., Chai H.B., Park S.Y., Kim D.S.H.L. (1999). Preparation of Amino Acid Conjugates of Betulinic Acid with Activity against Human Melanoma. Bioorg. Med. Chem. Lett..

[71] Wróblewska-Łuczka P., Cabaj J., Bąk W., Bargieł J., Grabarska A., Góralczyk A., Łuszczki J.J. (2022). Additive Interactions between Betulinic Acid and Two Taxanes in In Vitro Tests against Four Human Malignant Melanoma Cell Lines. Int. J. Mol. Sci..

[72] Decker T., Lohmann-Matthes M.-L. (1988). A Quick and Simple Method for the Quantitation of Lactate Dehydrogenase Release in Measurements of Cellular Cytotoxicity and Tumor Necrosis Factor (TNF) Activity. J. Immunol. Methods.

[73] Ates G., Vanhaecke T., Rogiers V., Rodrigues R.M. (2017). Assaying Cellular Viability Using the Neutral Red Uptake Assay. Cell Viability Assays: Methods and Protocols.

[74] Ghiƫu A., Pavel I.Z., Avram S., Kis B., Minda D., Dehelean C.A., Buda V., Folescu R., Danciu C. (2021). An In Vitro-In Vivo Evaluation of the Antiproliferative and Antiangiogenic Effect of Flavone Apigenin against SK-MEL-24 Human Melanoma Cell Line. Anal. Cell. Pathol..

[75] Ghițu A., Schwiebs A., Radeke H.H., Avram S., Zupko I., Bor A., Pavel I.Z., Dehelean C.A., Oprean C., Bojin F. (2019). A Comprehensive Assessment of Apigenin as an Antiproliferative, Proapoptotic, Antiangiogenic and Immunomodulatory Phytocompound. Nutrients.

[76] Danciu C., Muntean D., Alexa E., Farcas C., Oprean C., Zupko I., Bor A., Minda D., Proks M., Buda V. (2018). Phytochemical Characterization and Evaluation of the Antimicrobial, Antiproliferative and pro-Apoptotic Potential of Ephedra Alata Decne. Hydroalcoholic Extract against the MCF-7 Breast Cancer Cell Line. Molecules.

[77] Danciu C., Cioanca O., Hancianu M., Racoviceanu R., Muntean D., Zupko I., Oprean C., Tatu C., Paunescu V., Proks M. (2021). Botanical Therapeutics (Part II): Antimicrobial and In Vitro Anticancer Activity against Mcf7 Human Breast Cancer Cells of Chamomile, Parsley and Celery Alcoholic Extracts. Anti-Cancer Agents Med. Chem..

[78] Felice F., Zambito Y., Belardinelli E., Fabiano A., Santoni T., Di Stefano R. (2015). Effect of Different Chitosan Derivatives on in Vitro Scratch Wound Assay: A Comparative Study. Int. J. Biol. Macromol..

