# Supplementary material for: Enhanced Cytotoxicity and Antimelanoma Activity of Novel Semisynthetic Derivatives of Betulinic Acid with Indole Conjugation
Source: Plants (Basel). 2023 Dec 21;13(1):36. doi: 10.3390/plants13010036 (PMC10780819; doi:10.3390/plants13010036)
Supplement: Supplementary file 1 [file plants-13-00036-s001.zip › plants-2737901-supplementary.pdf]

Results

Anti-Migratory Activity Evaluation Employing the Scratch Assay Method

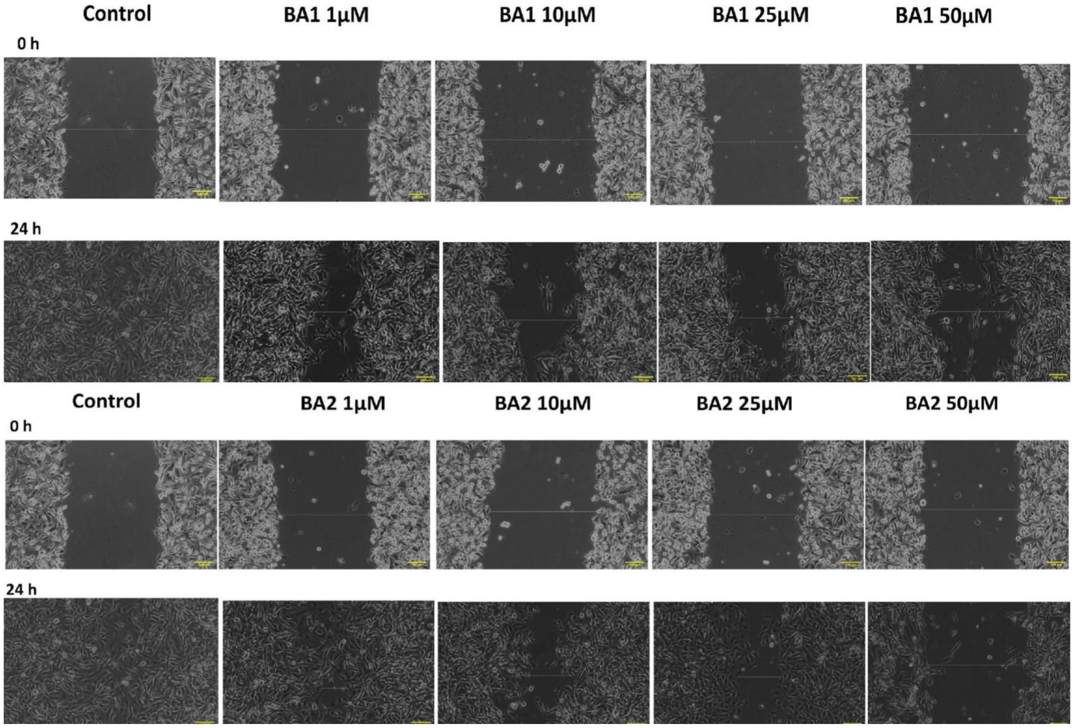

Figure S1. Anti-migratory activity of BA1 and BA2.

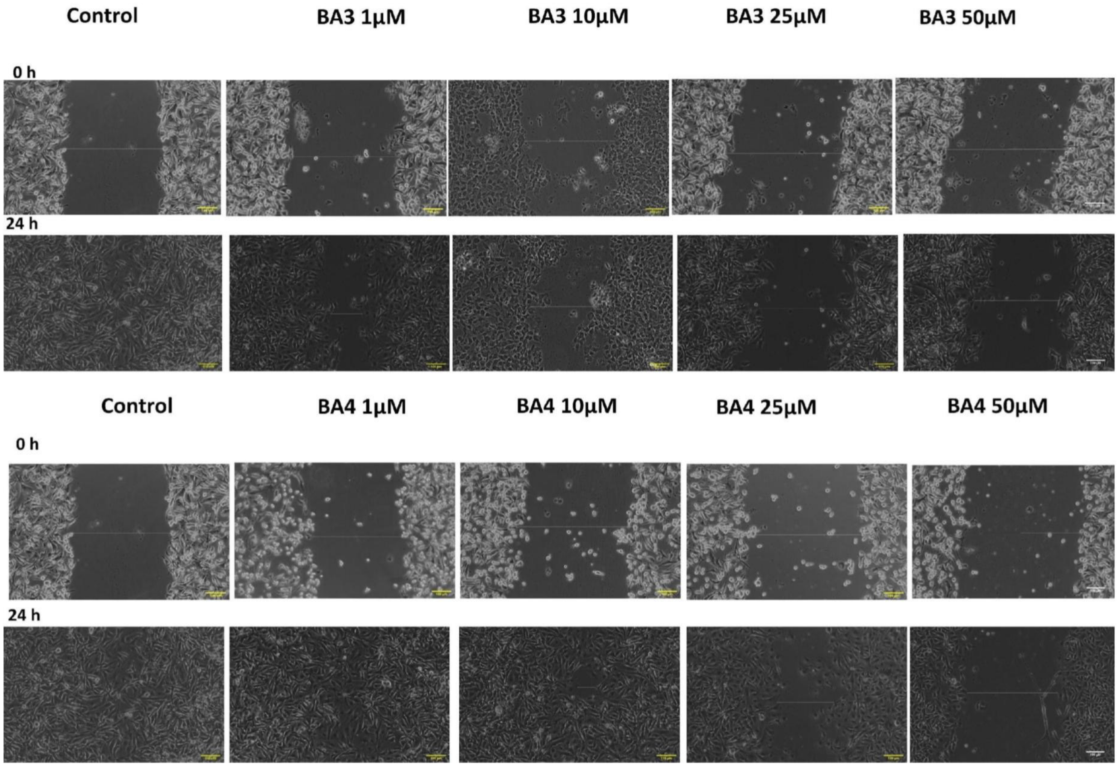

Figure S2. Anti-migratory activity of BA3 and BA4.

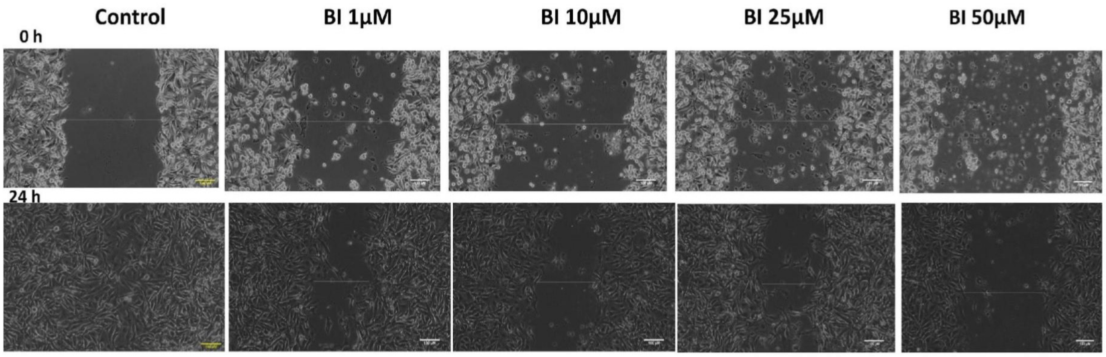

Figure S3. Anti-migratory activity of BI.
